# Early Use of Incisional Negative Pressure Wound Therapy in Pediatric Abdominal and Thoracic Surgery: A Single-Center Retrospective Study on Clinical and Economic Outcomes

**DOI:** 10.3390/children12111433

**Published:** 2025-10-23

**Authors:** Biagio Nicolosi, Felice Curcio, Marina Maffeo, Marika Di Leva, Mirco Gregorini, Emanuele Buccione, Riccardo Coletta

**Affiliations:** 1 Healthcare Professions Department, Meyer Children’s Hospital IRCCS, 50139 Florence, Italy; 2 Pediatric and Neonatal Surgery, University Hospital of Sassari, 07100 Sassari, Italy; felice.curcio@aousassari.it; 3 Pediatric Intensive Care Unit, Meyer Children’s Hospital IRCCS, 50139 Florence, Italy; marina.maffeo@meyer.it; 4 School of Human Health Science, University of Florence, 50139 Florence, Italy; 5 Pediatric Intensive Care Unit, Health Local Authority of Pescara, 65124 Pescara, Italy; emanuele.buccione@asl.pe.it; 6 Department NEUROFARBA, Meyer Children’s Hospital IRCCS, 50139 Florence, Italy

**Keywords:** negative pressure wound therapy, pediatric surgery, surgical wound complications, wound healing, economic outcomes, incisional NPWT

## Abstract

**Aim**: To evaluate the effectiveness and economic impact of early Incisional Negative Pressure Wound Therapy (iNPWT) in promoting surgical wound healing in pediatric patients undergoing abdominal and thoracic surgery. **Background**: Surgical wound complications, such as dehiscence and infection, are frequent in pediatric patients, especially in high-risk cases. Although iNPWT is increasingly used in surgical care, evidence supporting its efficacy in pediatric populations remains limited. **Methods**: This single-center, retrospective observational study analyzed 49 pediatric patients who underwent abdominal or thoracic surgery between January and December 2023. Patients received either intraoperative iNPWT (early application) or standard dressings. The outcomes assessed included time to complete wound healing, incidence of complications, pain levels, and healthcare costs. **Results**: Patients treated with early iNPWT showed significantly faster wound healing and fewer complications—particularly dehiscence and infections—compared to those receiving standard dressing. Pain perception did not significantly differ between groups. Although the initial costs of iNPWT were higher, overall costs were lower due to fewer complications and shorter hospital stays. **Conclusions**: Early iNPWT is a clinically effective and cost-efficient intervention for pediatric surgical patients at high risk of wound complications. However, limitations related to the retrospective design and small sample size suggest that prospective multicenter studies are needed to confirm these findings and support the development of standardized pediatric protocols.

## 1. Introduction

Surgical wound complications, such as dehiscence and infection, are relatively common in the pediatric population undergoing abdominal surgery [1,2,3]. According to main studies, the incidence of surgical site complications may range from 2% to 17% in pediatric patients undergoing major abdominal surgery, with increased risk in the presence of predisposing factors such as emergency surgery, repeat procedures, and comorbidities, as malnutrition, immunodeficiency, chronic diseases, prematurity, short bowel syndrome (SBS), intestinal diseases [1,4,5,6].

Negative Pressure Wound Therapy (NPWT) is a valuable approach to wound management, including pressure ulcers, acute wounds, burns, and surgical wounds. NPWT is particularly effective for treating complex injuries due to its mechanisms, which promote healing by removing extracellular fluid and exudate, enhancing local blood circulation, reducing edema, stimulating granulation tissue and cell proliferation, decreasing bacterial load, and facilitating the re-approximation of wound edges [7].

NPWT in pediatrics is largely used, including neonates and preterm, but the available evidence about methodologies is scarce [8]. Despite that, there is evidence supporting that supporting the use of NPWT in pediatric patients, although with some restrictions [6] . In children, devices and parameters are adapted to adult results, but it is important to establish technical values for tissue management of pediatric patients [8].

In children, devices and parameters are adapted based on the results of studies on the adult population, but it is important to establish technical values for tissue management of pediatric patients.

In the pediatric environment, the NPWT has several advantages, not only in clinics, but also in psychosocial. In fact, treatment safety and efficacy reduced the hospitalization time and the related risks, as infection, child and family stress, and pain [9]. Complications are rare and mostly manageable [8]. Recent studies are demonstrating its benefits in preventing surgical complications in children, particularly in high-risk populations [4].

Comparative research is needed to define the effectiveness of treatment in terms of prevention and healing, advantages and disadvantages, pediatric-specific instructions, and the limits of the NPWT in children [8].

The aim of this study is to analyze the effectiveness of incisional NPWT (iNPWT) in terms of total healing time of the surgical wounds in the pediatric population.

## 2. Materials and Methods

This retrospective study included 49 pediatric patients divided into two groups: 28 children who received early iNPWT at the time of surgery (Group 1), and 21 who were treated with tradional dressing at the time of surgery (Group 2). Inclusion criteria were children aged 0–18 years undergoing abdominal or thoracic surgery who required NPWT, with informed consent obtained as appropriate. This study was conducted in a single tertiary pediatric referral hospital, between 1 January and 31 December 2023, ensuring consistent protocols and follow-up. The authors adhered to the ‘Strengthening the Reporting of Observational Studies in Epidemiology (STROBE) statement’ guidelines [10].

### 2.1. Inclusion Criteria

Patients were divided into two groups according to the timing of NPWT initiation. Group 1 consisted of children at high risk of wound complications, identified intraoperatively based on risk factors such as multi-operated surgical sites, tissue fragility, compromised vascularization, or surgeon-identified local conditions. These patients received iNPWT immediately after wound closure, while group 2 included children initially managed with standard dressings.

Exclusion criteria included surgery without iNPWT/NPWT, lack of informed consent, or incomplete data. Although allocation was based on surgeon judgment, the criteria were standardized as described. Nevertheless, some subjectivity may have introduced a selection bias, potentially leading to greater clinical complexity in Group 2.

Furthermore, inclusion in the study required written informed consent from the parents or legal guardians for children under 7 years of age, and additionally from the child for those aged 7 years or older. In cases of complications, some children were discharged and managed through outpatient clinic visits until full recovery was achieved.

### 2.2. Data Collection

Data were collected from computerized clinical records and entered into an electronic database (REDCap^®,^ Vanderbilt University, Nashville, TN, USA) consisting of 21 elements. Information included sex, age, primary diagnosis, treatment duration and type, treatment specifics, outcomes, and interventions. All recorded timeframes were expressed in days.

For each group, we recorded the type of foam used, the suction pressure applied (adjusted according to the child’s weight), the frequency of dressing changes, and any complications (infection, dehiscence, and pain), classified using the Sandy Grading System [11] and the TIMERS classification [12]. We also documented how complications were managed, recovery times, and the average pain scores observed during treatment. Pain was assessed using age-appropriate tools: the Neonatal Pain, Agitation, and Sedation Scale (N-PASS) [13] for children aged 0–60 days, the Face, Legs, Activity, Cry, Consolability Scale (FLACC) [14] for children aged 60 days to 7 years, and the Visual Analog Scale (VAS) [15] for children aged 7 years and older.

Sociodemographic data, such as admission diagnosis, sex, age, weight, and length of hospital stay, were also recorded. Additionally, we collected the technical characteristics of the negative pressure therapy at initiation, including the suction pressure and type of foam used.

Lastly, we analyzed the cost of each treatment in detail, including the expense of all dressings used throughout the treatment period (2023). Data were extracted by trained clinical researchers who were not involved in patient care and were blinded to group assignment during outcome evaluation.

### 2.3. Primary and Secondary Outcomes

The primary outcome of the study was the time to complete surgical wound healing, expressed in days from the application of iNPWT or standard dressing to full epithelialization.

The secondary outcomes included: the occurrence of postoperative complications (dehiscence and infection), pain intensity measured using validated pediatric scales, frequency of dressing changes, and the cost analysis of the entire treatment.

### 2.4. Ethical Concerns

This study was approved by the Ethics Committee of the Tuscany Region—Pediatric (approval number 45/2024). Informed consent was obtained from all participants involved in the study. Patients enrolled in the study did not receive any additional medical, pharmacological, or behavioral interventions beyond those routinely administered.

### 2.5. Statistical Analysis

Descriptive statistics were reported as appropriate, following the assessment of normality for continuous variables using the Shapiro–Wilk test. To assess the adequacy of the sample size used in this study, a post hoc power analysis was conducted using G*Power software (version 3.1, Heinrich Heine University, Düsseldorf, Germany) [16]. For the main comparison, Cohen’s d effect size was calculated. Although the primary analysis was based on a Kaplan–Meier survival analysis using the log-rank test, a two-tailed independent samples *t*-test was used as a conservative approximation for power estimation, treating healing time as a continuous variable. Frequency and percentage were provided for qualitative variables, while mean and standard deviation (SD) were calculated for quantitative variables with a normal distribution. For quantitative variables with a non-normal distribution, the median and interquartile range (IQR) were reported.

Statistical tests were conducted to compare the baseline characteristics of the two groups to ensure their comparability. For quantitative variables, an independent *t*-test or the Mann–Whitney U test was applied as appropriate. Comparisons between two groups for continuous variables were performed using the independent samples *t*-test when assumptions of normality were met. For categorical variables, the Chi-square test or Fisher test was used. Kaplan–Meier survival analysis was performed to evaluate treatment outcomes associated with early complete healing. To compare pain levels during dressing changes, the Kruskal–Wallis test was employed.

Lastly, a multivariate linear regression analysis was conducted to identify independent variables associated with changes in time to complete healing. Regression results are presented as unstandardized and standardized coefficients, along with their 95% confidence intervals. The model’s coefficient of determination was also reported.

Given that the primary outcome was time-to-event (complete healing), a Cox proportional hazards model may have provided a more suitable analytical approach. However, a linear regression model was used in this study due to the nature of the available data and to facilitate the interpretation of continuous time differences.

Statistical significance was set at a *p*-value < 0.05. All statistical analyses were performed using IBM© SPSS software, version 22.0.

## 3. Results

Among the 49 children included in the study, 28 (57.1%) were male. Group 1 (iNPWT at surgery) included 28 patients (57.1%), while Group 2 (standard dressing at surgery) included 21 patients (42.9%). A total of 43 children (87.8%) underwent abdominal surgery, and 28 (57.1%) were treated with iNPWT. The mean age was 74.59 months (SD 71.96), and the mean weight was 22.91 kg (SD 19.19).

The main admission diagnoses are summarized in Table 1.

A total of six pediatric surgeons were involved in the procedures. Four surgeons operated in both groups, while two were responsible for cases exclusively in Group 1. No systematic differences in surgeon distribution across groups were identified.

The median length of stay (LOS) was 22 days [IQR 10.5–33]. No significant difference in length of stay was observed between Group 1 (20 days) and Group 2 (24 days), with F = 0.851 and *p* = 0.361. The baseline characteristics of both groups are detailed in Table 2.

The effect size (Cohen’s d) was automatically calculated by G*Power using the pooled standard deviation. To perform the power analysis, the following observed parameters were used: number of patients and mean healing time (in days) with related standard deviations of both groups.

**Figure 1:**
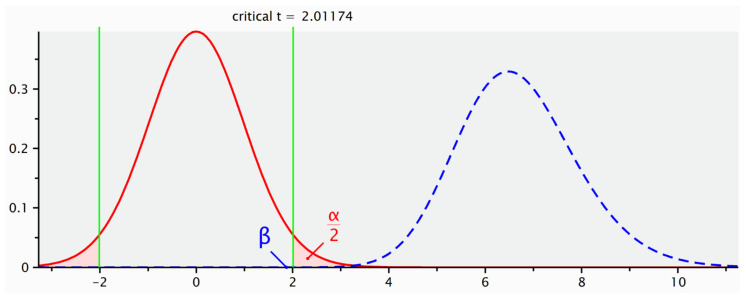
Statistical Power Analysis for Sample Size Estimation. Post hoc analysis demonstrated a statistical power of 85.4% (Cohen’s d = 1.896), confirming adequate sample size to detect significant differences in healing time between groups. The green lines delimit the critical regions of the t-test, that is, the situations in which no real difference exists between the groups being compared.

The analysis showed a statistical power of 85.4%, based on a large observed effect size (Cohen’s d = 1.896). These findings indicate that the sample size was adequate to detect a statistically significant difference between the two treatment conditions. The computed statistical power (1 − β) was 85.4%, assuming an alpha level of 0.05 (Figure 1).

### 3.1. Postoperative Complications

After surgery, the average wound length was 7.15 cm (SD 2.26). A statistically significant difference was observed between the groups, with a longer mean wound length in Group 2 (7.95 cm, SD 2.55) compared to Group 1 (6.43 cm, SD 1.75) (t = 2.50, *p* = 0.017).

Local complications were noted in 21 children (42.9%), including wound dehiscence in 34.7% (17/49) and postoperative wound infection in 8.2% (4/49). The mean time to onset of local complications was 4.38 days (SD 1.91). All complications occurred in Group 2, with a statistically significant difference (*p* < 0.001) as determined by the Chi-square test.

### 3.2. Wound Management

No significant difference was observed in the frequency of dressing changes between the groups. The mean interval between dressing changes was 67.43 h (SD 12.28) in Group 2 compared to 72 h in Group 1 (F = 3.9, *p* = 0.054). Additionally, no significant difference in pain levels was noted between the groups (*p* = 0.658), as determined by the Kruskal–Wallis test.

In Group 1, the wound healing process proceeded without the need for treatment modifications. Conversely, Group 2 required adjustments due to complications previously described, including the use of silver gelling fiber (1/21, 4.76%), bacteria-binding dressing with hydrogel (2/21, 9.52%), and NPWT (18/21, 85.71%).

**Figure 2:**
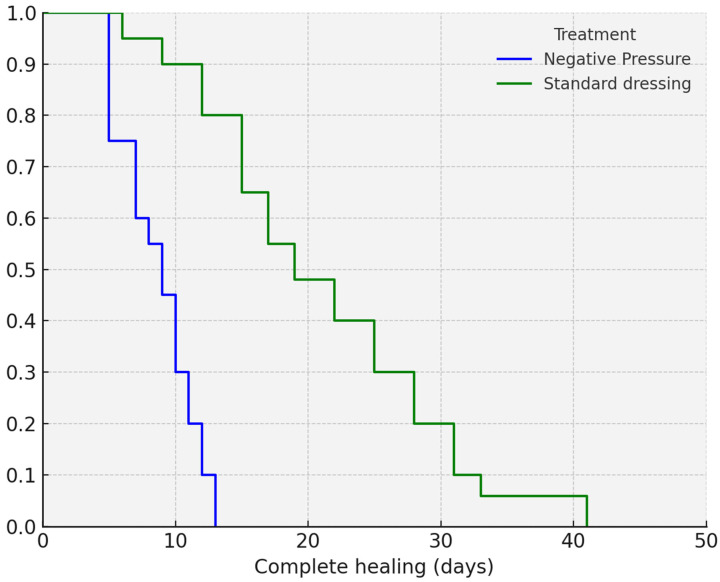
Kaplan–Meier curves showing complete healing time across treatment groups. Group 1: Negative pressure; Group 2: Standard dressing. Comparison of complete wound healing over time between Group 1 (early iNPWT application) and Group 2 (standard dressing ). The survival curves show significantly faster healing in Group 1 (*p* < 0.001).

The mean time to complete healing was significantly shorter in Group 1. According to Kaplan–Meier analysis, complete healing was achieved in 8.78 days (95% CI: 7.82–9.74) in Group 1, compared to 19.42 days (95% CI: 15.98–22.87) in Group 2 (*p* < 0.001). The Kaplan–Meier analysis is shown in Figure 2.

Additionally, a multiple linear regression analysis was conducted to examine the association between independent variables and the time to complete healing (Table 3).

The analysis revealed that time to complete healing was not significantly associated with patients’ age (*p* = 0.75), weight (*p* = 0.8), length of hospital stay (*p* = 0.54), wound length (*p* = 0.61), or suction pressure (*p* = 0.88) during negative pressure therapy. The regression model showed an R^2^ of 0.12, indicating that only 12% of the variance in healing time was explained by the included variables.

### 3.3. Follow-Up

Fourteen percent (7/49) of the participants required wound treatment after hospital discharge. All seven patients were initially treated with standard dressings (*p* = 0.001, according to the Chi-square test). Once approximately 60% wound healing was achieved and the wound characteristics met the TIMERS criteria for outpatient management (Atkin et al., 2019), the children were discharged [17]. They were periodically reassessed, on average every 3 days, at the complex injuries clinic until complete healing was achieved.

### 3.4. Negative Pressure Therapy Technical Details

Differences were observed in the type of foam used. In the iNPWT group, the most commonly used foam was White Foam^®^, whereas in patients who were initially treated with standard dressings and later switched to NPWT, Granu Foam Silver^®^ was the most commonly used (*p* = 0.004, according to the Chi-square test). Regarding suction pressure, a lower pressure was applied in the iNPWT group, with a mean pressure of −60.71 mmHg (SD 19.75), compared to −96.05 mmHg (SD 38.42) in the standard dressing group in which NPWT was subsequently applied (F = 17.13, *p* < 0.001).

### 3.5. Healthcare Spending

A detailed cost analysis was conducted to compare the average healthcare spending associated with each group. Considering the cost of renting the device (approximately €22 per day [$23.08]) and the cost of advanced dressings (approximately €4.72 per replacement [$4.96]), a higher average cost was observed in group 2 compared to group 1, with average costs of €370.85 [$389.13] and €193.28 [$202.81], respectively.

## 4. Discussion

iNPWT has proven to be an effective strategy for treating and preventing surgical wound complications in pediatric care [4,9]. The results of this study demonstrate that early application of iNPWT in pediatric patients undergoing abdominal and thoracic surgery significantly reduces healing time compared to treatment with standard dressings. Pérez-Acevedo et al. (2024) highlighted the effectiveness of iNPWT in preventing surgical site complications and promoting faster healing by approximating incision edges and reducing the traction and tension forces caused by patient movement [4,18]. These findings align with the growing body of evidence on the benefits of iNPWT in pediatric surgical patients [4].

As reported by Visser et al. (2017) [19], iNPWT helps reduce surgical wound complications and hospital length of stay. In our study, although the overall hospital stay length did not significantly differ between groups, the underlying causes varied. In Group 2, prolonged stays were attributable to wound complications, whereas in Group 1, duration was consistent with standard postoperative recovery. This distinction highlights the potential of iNPWT to indirectly mitigate prolonged hospitalization due to surgical site complications. Reducing the number of days spent in hospital remains one of the main objectives for reducing the onset of complications related to hospitalization, such as the onset of skin injuries [20]. It should be considered that a longer wound length and a higher proportion of thoracic procedures in Group 2, although not statistically significant, may have contributed to worse outcomes.

Additionally, 14% of patients in Group 2 required outpatient wound management after discharge, emphasizing the longer healing trajectory . This approach has shown particular benefit for high-risk patients, especially those with multiple abdominal and thoracic surgical sites or local risk factors identified during surgery. The use of iNPWT is gaining traction in pediatric surgical practice and has been integrated into several clinical recommendations and best practice guidelines [5,21]. A meta-analysis by Groenen et al. (2023) provides strong evidence supporting the efficacy of iNPWT in reducing surgical site complications [22].

In our study, no wound complications were observed in Group 1 (iNPWT), while Group 2 experienced complications, including dehiscence and infection. Infections in patients treated with standard dressings reached statistical significance, corroborating findings from White et al. (2022) [18]. Our study contributes to this growing evidence by demonstrating that early use of iNPWT not only prevents complications but also facilitates a simpler clinical course, minimizing the need for advanced wound care strategies postoperatively.

Pain levels, assessed using validated pediatric scales (N-PASS, FLACC, VAS), did not differ significantly between groups, suggesting that iNPWT is well tolerated and does not negatively impact patient comfort or quality of life.

The economic analysis revealed that the iNPWT approach incurred lower costs than the standard dressing approach, consistent with the conclusions of Pérez-Acevedo et al. (2024) [4]. The study demonstrated that, although the initial costs of iNPWT were higher, the overall costs associated with the treatment of surgical wound complications, including dressing changes and reoperations, were significantly lower. These results underscore the cost-effectiveness of early iNPWT, as it minimizes the economic burden associated with prolonged treatment, dressing supplies, and potential reoperations [4]. However, to fully realize the benefits of iNPWT in pediatric care, greater training for healthcare professionals and standardized protocols are necessary.

This study has several limitations. First, its retrospective design may introduce bias and limit the accuracy or completeness of recorded data. Second, the lack of randomization between groups could have led to confounding, especially considering the clinical criteria used to assign patients to iNPWT. Furthermore, the heterogeneity in surgical type may have affected group comparability and statistical reliability. Lastly, subjectivity in surgical decision-making could have introduced a selection bias.

Although the study was not preceded by a formal sample size calculation, the post hoc analysis revealed a power of 85.4%, which exceeds the conventional threshold of 80% typically required for clinical research [23]. This result, together with the very large effect size, supports the statistical robustness of the findings and suggests that the observed difference in healing time between groups is unlikely to be due to chance. It is important to acknowledge that the post hoc analysis was based on a *t*-test approximation rather than a survival-specific test such as the log-rank and thus does not account for censored data. This represents a methodological limitation. However, the high power observed reinforces the hypothesis of a clinically relevant benefit associated with the experimental treatment.

Further prospective, multicenter studies with larger sample sizes are needed to confirm these findings and provide more insight into the impact of iNPWT on the quality of life for patients and their families.

Despite these limitations, our findings provide valuable insights into the clinical and economic benefits of early iNPWT in pediatric surgical care. Further prospective, multicenter trials are essential to validate these results and to inform standardized protocols aimed at improving outcomes for high-risk pediatric patients.

## 5. Conclusions

This retrospective study suggests that incisional iNPWT may be a cost-effective strategy for preventing surgical complications, such as wound dehiscence and infection, in pediatric patients undergoing abdominal and thoracic procedures. The findings indicate that early use of iNPWT is associated with a reduced risk of postoperative complications, particularly dehiscence, and a shorter time to complete wound healing, without increasing the pain experienced by patients.

Our findings support the early use of iNPWT as a clinically effective and economically advantageous intervention for preventing postoperative complications in pediatric surgical patients.

## Figures and Tables

**Table 1:** Admission diagnoses of the pediatric surgical population (n = 49). Data are expressed as absolute frequency (n) and percentage (%).

Admission Diagnosis	n (%)
Recanalization intervention	21 (42.9)
Mastectomy	3 (6.1)
Necrotizing enterocolitis	3 (6.1)
Intestinal malrotation	2 (4.1)
Pectus excavatum	2 (4.1)
Pancreatitis	2 (4.1)
Short bowel syndrome	2 (4.1)
Other conditions	14 (28.5)
Total	49 (100)

**Table 2:** Sample’s characteristics according to the timing of iNPWT. SD, standard deviation; N, absolute frequency; Age, months; Weight, kilograms; Wound length, centimeters; Frequency changes, days; post-operative complication onset, days; *: according to Chi-Square test; **: according to Fisher test; bold values, statistically significant.

Factor		Group 1 [SD] (n)	Group 2 [SD] (n)	F	*p* Value
Gender	Female	(14)	(7)		0.382 *
	Male	(14)	(14)		
Age		59.57 [57.15]	94.61 [85.33]	2.961	0.092
Weight		19.04 [15.37]	28.07 [22.73]	2.751	0.104
Wound Length		6.43 [1.75]	7.95 [2.55]	6.125	**0.017**
Frequency Changes		72.00 [0.00]	67.43 [12.28]	3.906	0.054
Post-operative complication onset		/	4.38 [1.91]	148.380	<0.001
Site	Abdomen	(27)	(16)		0.072 **
	Chest	(1)	(5)		
Local Complications	Yes	(0)	(21)		<0.001 *
	No	(28)	(0)		

Demographic and clinical characteristics of pediatric patients in Group 1 (early iNPWT) and Group 2 (standard dressing), with comparison of variables such as wound length, complication onset, and local complications. Statistically significant values are highlighted in bold.

**Table 3:** Independent variables predicting time to complete healing. R^2^, coefficient of determination; B, unstandardized coefficient; β. Standardized coefficient: 95% CI: confidence interval.

Factor	B	β	95% CI		*p* Value
Age	0.036	0.334	−0.196	0.269	0.755
Weight	−0.105	−0.259	−0.958	0.747	0.804
Length of stay	0.065	0.319	−0.001	0.131	0.054
Wound length	1.173	0.341	−0.057	2.403	0.061
Suction pressure	−0.008	−0.035	−0.122	0.106	0.884
				R^2^: 0.224	
				Adjusted R^2^: 0.124	

Multivariate linear regression analysis assessing the influence of clinical and treatment-related variables on healing time. None of the included predictors showed statistically significant associations (*p* > 0.05). Adjusted R^2^ = 0.124.

## Data Availability

The datasets generated and/or analyzed during the current study are available from the corresponding author on reasonable request.
